# Regeneration of Recombinant Antigen Microarrays for the Automated Monitoring of Antibodies against Zoonotic Pathogens in Swine Sera

**DOI:** 10.3390/s150202614

**Published:** 2015-01-23

**Authors:** Verena K. Meyer, Catharina Kober, Reinhard Niessner, Michael Seidel

**Affiliations:** Chair for Analytical Chemistry and Institute of Hydrochemistry, Technische Universität München, Marchioninistrasse 17, 81377 Munich, Germany; E-Mails: verena.meyer@mytum.de (V.K.M.); kober.catharina@gmail.com (C.K.); reinhard.niessner@ch.tum.de (R.N.)

**Keywords:** chemiluminescence microarrays, indirect microarray immunoassays, regeneration, automated analysis platforms, food safety, zoonotic pathogens, recombinant antigen, meat processing, serology

## Abstract

The ability to regenerate immobilized proteins like recombinant antigens (rAgs) on surfaces is an unsolved problem for flow-based immunoassays on microarray analysis systems. The regeneration on microarray chip surfaces is achieved by changing the protein structures and desorption of antibodies. Afterwards, reactivation of immobilized protein antigens is necessary for reconstitution processes. Any backfolding should be managed in a way that antibodies are able to detect the protein antigens in the next measurement cycle. The regeneration of rAg microarrays was examined for the first time on the MCR3 flow-based chemiluminescence (CL) microarray analysis platform. The aim was to reuse rAg microarray chips in order to reduce the screening effort and costs. An antibody capturing format was used to detect antibodies against zoonotic pathogens in sera of slaughtered pigs. Different denaturation and reactivation buffers were tested. Acidic glycine-SDS buffer (pH 2.5) and 8 M guanidinium hydrochloride showed the best results in respect of denaturation efficiencies. The highest CL signals after regeneration were achieved with a carbonate buffer containing 10 mM DTT and 0.1% BSA for reactivation. Antibodies against *Yersinia* spp. and hepatitis E virus (HEV) were detected in swine sera on one immunochip over 4 days and 25 measurement cycles. Each cycle took 10 min for detection and regeneration. By using the rAg microarray chip, a fast and automated screening of antibodies against pathogens in sera of slaughtered pigs would be possible for zoonosis monitoring.

## 1. Introduction

Zoonoses are infectious diseases that can be transmitted from animals to humans [1]. Zoonotic pathogens in meat and meat products are relevant sources for human infections [2]. Emerging meat-borne pathogens besides *Salmonella* spp. and *Toxoplasma* spp. are e.g., HEV, *Campylobacter* spp., and *Yersinia* spp. [3–8]. Zoonotic pathogens in meat have to be controlled by a complete, continuous farm-to-fork system [9], such as in Sweden [10] or in Denmark [11,12]. Bacteriological cultivation methods and serological tests by indirect enzyme-linked immunosorbent assays (ELISA) [13,14] are established as well as immunochromatographic assays [15,16] and microparticle-based assays [17,18]. However, ELISA tests for other zoonotic pathogens besides *Salmonella* spp. are not yet accepted for routine analysis of meat juice [19], costs and assay time per sample have to be reduced, sampling and analysis processes have to be adapted to use by unskilled personal, and bioanalytical systems have to be linked to traceability systems [20,21]. A complete monitoring for all relevant zoonotic pathogens at slaughter is only manageable by fast and fully automated multi-analyte immunoassays. Therefore, research on microarray-based analysis systems is in high demand. The ability to regenerate rAg microarrays is not yet studied, although this is necessary to become accepted as a routine hygiene monitoring method for food safety.

Multi-analyte assays are available on analysis platforms like the Luminex, Randox, or MCR3 platforms [22]. The MCR3 used in this study is an automated analysis platform performing flow-based CL microarrays [23]. An immunochip was developed that is able to detect antibodies against emergent zoonotic pathogens like *Yersinia* spp. and HEV in swine sera by affinity binding to recombinant antigens [24]. On the MCR3, the regeneration of microarray chips has only been demonstrated so far for indirect competitive microarray immunoassays [25] with small organic molecules like antibiotics [26], phycotoxins [27], mycotoxins [28], or carbohydrates [29] immobilized on the surface. Acidic regeneration buffers are flushed over the microfluidic flow cell that contain denaturation agents like SDS. The affinity binding between antibody and immobilized organic molecule is disturbed and the labeled antibody can be removed by hydrodynamic flow. The regeneration of rAg microarrays is more challenging because the first denaturation step deactivates the functionality of the protein as well. A second reactivation step is necessary that induces backfolding of the proteins on the chip surface. Inefficient regeneration of CL microarrays is characterized by remaining HRP activity on the microarray chip after the denaturation processes or reduced CL signals after reactivation.

The aim of the present study was to show the proof of concept of regenerating recombinant antigens on the MCR3. *Yersinia* spp. and HEV positive sera of slaughtered pigs were used to examine the regeneration efficiency of three different recombinant antigens. A measurement strategy was established to determine the efficiency of different denaturation and reactivation buffers.

## 2. Experimental Section

### 2.1. Chemicals and Materials

Absolute ethanol 99.8%, bovine serum albumine (BSA), dipotassium hydrogen phosphate, disodium hydrogen phosphate, sodium hydroxide, dithioerythritol (DTE), dithiothreitol (DTT), 3-glycidyloxypropyl trimethoxysilane (GOPTS), guanidinium hydrochloride (GuHCl), hydrogen chloride (37%), methanol, Pluronic^®^ F-127, poly(ethylene glycol) diglycidyl ether (diepoxy-PEG, M_N_ = 500), potassium dihydrogen phosphate, sodium azide, sodium carbonate, sodium chloride, sodium dodecyl sulfate (SDS), sodium hydrogen carbonate, fuming sulfuric acid, D-(+)-trehalose dihydrate, tris(hydroxymethyl)aminomethane Sigma 7–9^®^ (TRIS), Tween^®^-20 and urea were obtained from Sigma-Aldrich (Taufkirchen, Germany). 3-(*N*-morpholino)-propanesulfonic acid (MOPS PUFFERAN^®^) and Menzel glass slides (76 mm × 26 mm × 1 mm) were obtained from Carl Roth (Karlsruhe, Germany). Hellmanex was obtained from Hellma GmbH (Mannheim, Germany). Jeffamine^®^ ED-2003 polyetheramine (DAPEG) was obtained from Huntsman Belgium BVBA (Everberg, Belgium) as a gift. WESTAR supernova ELISA luminol and hydrogen peroxide were purchased from Cyanagen (Bologna, Italy). The ARcare 90106 adhesive film was obtained from Adhesive Research Ireland (Limerick, Ireland). The poly(methyl methacrylate) support for the chip was produced in our workshop. 384-well PP flat bottom microtiter plates (MTPs) were obtained from Greiner GmbH (Frickenhausen, Germany).

### 2.2. Prepared Aqueous Buffer Solutions

Aqueous buffer solutions were prepared with deionized and treated water by a Milli-Q plus 185 system (Millipore, Schwalbach, Germany).

Phosphate buffer saline (PBS): 10 mM potassium dihydrogen phosphate, 70 mM dipotassium hydrogen phosphate, 145 mM sodium chloride were adjusted with hydrochloric acid to pH 7.4. PBST contained additional 0.05% (v/v) Tween^®^-20 and was used as running buffer on the MCR3, for dilution of antibodies, sera, and BSA.
Blocking buffer: Aqueous buffer that contained 1 M TRIS, 150 mM sodium chloride, and 0.05% (v/v) Tween^®^-20. The blocking buffer was adjusted with hydrochloric acid to pH 7.8.
MOPS buffer: This buffer was used for storage of rAgs and contained 20 mM MOPS (pH 7.2), 10 mM DTE, and SDS (0.05% (w/v) for ORF2C-gt1, 0.02% (w/v) for ORF2C-gt3, and 0.01% (w/v) for YopD).
8 M GuHCl: This buffer solution contained 8 M guanidinium hydrochloride and the pH was 5.2.
6 M urea: This buffer solution contained 6 M urea and the pH was 6.4.
MDSB: This buffer solution contained 20 mM MOPS, 10 mM DTT, and 0.1% (w/v) SDS. The pH was 4.8.
G-SDS: This buffer solution contained 100 mM glycine, 100 mM NaCl, and 1% (w/v) SDS. The pH was adjusted to pH 2.5 with 37% (v/v) HCl.
Reactivation buffer: This buffer solution contained 15 mM Na_2_CO_3_, 35 mM NaHCO_3_, 10 mM DTT, and 0.01% (v/v) Tween^®^-20 (RB1) or 0.1% (w/v) BSA (RB2). The pH was 9.5.

### 2.3. Surface Modification of Glass Slides

Glass slides were treated and functionalized according to procedures described elsewhere [30]. Briefly, microscope glass slides were cleaned with 2% Hellmanex solution and activated in methanol/hydrochloric acid (1:1) and subsequently in fuming sulfuric acid. Two hydroxyl-terminated glass slides were silanized for 3 h at RT with 600 μL GOPTS by forming a sandwich. Silanized glass slides were separated in ethanol and cleaned extensively by sonication in ethanol, methanol, and ethanol for 15 min, respectively. After drying under a nitrogen stream, two glass slides were coated with 600 μL molten DAPEG in smelter at 98 °C for 15 h by forming a sandwich. The resulting diamino-PEG-coated glass slides were washed extensively with water, sonicated two times for 15 min each in water, and finally dried under a nitrogen flow. The diamino-PEGylated chips were stored under vacuum for a maximum time of six weeks. Coated glass slides were activated with diepoxy-PEG before rAgs were immobilized. Therefore, 600 μL diepoxy-PEG were dispensed on one and covered with another coated glass slide. Each sandwich was incubated for 15 h at 100 °C. After cleaning with methanol by means of sonication two times for 15 min each and drying under nitrogen, the epoxy-activated PEG-coated glass slides were directly used for the contact printing process.

### 2.4. Preparation of rAg Microarrays

The preparation process of rAg microarrays was performed as described elsewhere [24]. The recombinant antigens of HEV and *Yersinia* spp. were provided by Mikrogen GmbH (Neuried, Germany). Stock solutions of rAgs ORF2C-gt1 (1.06 mg/ml) and ORF2C-gt3 (2.06 mg/mL) were supplied in MOPS buffer that contained 0.05% and 0.02% SDS, respectively. The stock solution of rAg YopD (0.28 mg/mL) consisted of MOPS buffer solution containing 0.01% SDS. The rAg solutions were stored in small aliquots at −80 °C before use. The rAg microarray was produced by contact printing using the BioOdyssey Calligrapher MiniArrayer from Bio-Rad Laboratories GmbH (Munich, Germany) and solid pin SNS 9 from ArrayIt (Sunnyvale, CA, USA). Anti-swine antibodies (goat) and anti-goat antibodies (rabbit) were purchased from KPL (Gaithersburg, MD, USA) as positive controls. The antibodies were diluted to a concentration of 1 mg/mL in PBS (pH 7.4) and contained 0.005% Pluronic F-127 and 10% trehalose. rAgs, positive and negative control were stored in 384-MTPs (polypropylene) in the contact printing unit at 20 °C and 50% humidity. Each immunochip had two separate flow cells with a distance of 11.75 mm. Two clusters were set on one microarray chip with a grid spacing of 1300 μm for the columns and 1100 μm for the rows, respectively. Each solution was spotted in 5 replicates. Slides were cooled to 20 °C during the spotting process and the humidity in the spotting chamber was set to 50%. After spotting, immunochips were incubated for 15 h at 25 °C and 50% humidity. Any remaining binding sites were inactivated by gently shaking the slides in blocking buffer for 15 min, followed by a 30 min incubation in 1% (w/v) BSA in PBST. Finally, the slides were rinsed three times with PBST and cleaned by shaking in PBST for 15 min.

After drying under a continuous nitrogen flow, the glass slides were connected with a PMMA plastic carrier presenting in- and outlets by use of a double-sided adhesive foil that forms two microfluidic measuring channels of one microarray chip. Each flow cell had a volume of 43.3 μL and a microarray area of 12 mm × 8 mm. The completed microarray chip was either filled with PBST and stored at 4 °C or used directly for measurements on the MCR3 system.

### 2.5. Serological Measurements with rAg Microarrays on MCR3

**Figure 1.**
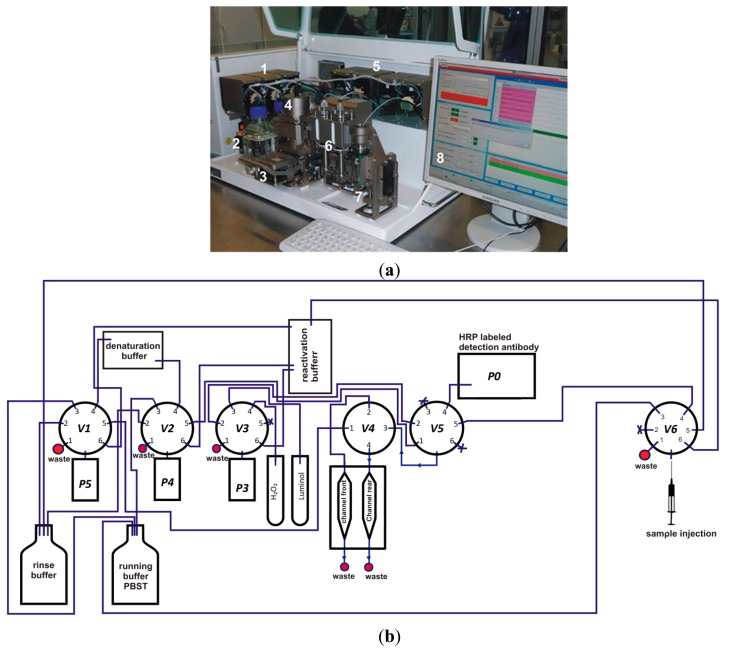
Image of the MCR3 equipped with following components (**a**): pumping unit for buffer delivery (1), reservoirs for buffer solutions (2), loading unit for immunochips (3), detection unit with CCD camera (4), valves for the processing of CL-MIA (5), antibody syringes (6), sample syringe (7), and computer-controlled software for automated processes and data evaluation (8). Fluidic set-up of the MCR3 for the performance of antibody capturing assays (**b**).

The measurement program for the analysis of antibodies in sera of slaughtered pigs is described elsewhere in detail [24]. The microarray chip was placed in the detection unit of the MCR3 from GWK Präzisionstechnik GmbH (Munich, Germany) and was connected with the flow system by closing the upper part of the detection unit (see Figure 1).

**Figure 2.**
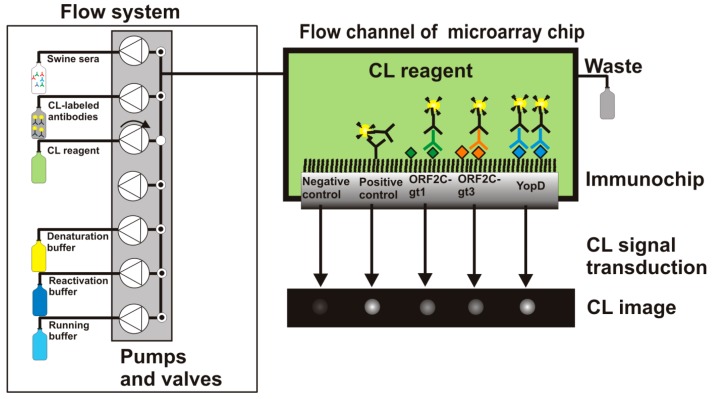
Scheme for flow-based CL microarray immunoassay to detect antibodies in swine sera with rAg microarrays on the MCR3 analysis platform. Swine sera, CL-labeled antibodies, CL reagent, denaturation buffer, and reactivation buffer are consecutively pumped through the flow channel of the microarray chip.

More information about the MCR3 microarray analysis platform is given elsewhere [23]. Before the measurement was started, all needed buffers and CL reagents were prepared and loaded for automated supply. A 50-mL glass syringe was connected with the MCR3 that contained anti-swine IgG-HRP conjugate (0.5 mg/mL) diluted 1:500 in PBST. 1-L glass bottles of running buffer (PBST), denaturation buffer, and reactivation buffer were connected to the defined tubing connectors of the MCR3. The CL reagents luminol and hydrogen peroxide were placed in 10-mL reagent tubes. The computer-controlled protocol of the flow-based CL antibody capturing microarray immunoassay is schematically shown in Figure 2 and can be summarized as follows: 1000 μL of diluted serum (1:100 in PBST) were injected in the fluidic system using a disposable syringe. With a high flow rate of 100 μL/s, 100 μL were injected to fill the tube with sample solution and 200 μL of the sample were pumped to the measurement channel. 700 μL were flushed over the microarray chip at a flow rate of 10 μL/s. Subsequently, 1000 μL of running buffer were pumped over the chip at a flow rate of 10 μL/s and 2000 μL of running buffer at a flow rate of 500 μL/s. Afterwards, the detection antibody solution was injected. First, 200 μL of detection antibody were dispensed at a flow rate of 100 μL/s, then 800 μL at 10 μL/s. The fluidic system was rinsed extensively (2000 μL running buffer, 500 μL/s) before a mixture of both CL substrates (200 μL each) was injected at a flow rate of 150 μL/s. The flow was stopped and an image was taken by the CCD camera for 60 s. The measurement process took 7 min. 2D images of the flow-based CL microarray reaction were taken with a 16-bit CCD camera (2 × 2 pixel binning mode, 696 × 520 pixels). A background image was taken from each immunochip prior the first measurement. This image was automatically subtracted from each measuring image.

### 2.6. Regeneration Experiments

The fluidic system was extensively rinsed with running buffer (5 mL, 400 μL/s) before the regeneration of rAg microarrays was started. Subsequently, the rAg microarray chip was rinsed with different denaturation buffer (5 mL, 400 μL/s) and reactivation buffer (5 mL, 400 μL/s). For more efficient treatment, each buffer was moved slowly five times forwards and backwards (100 μL, 10 μL/s). A CL image was taken after denaturation and reactivation, respectively, by injection of CL reagents (200 μL each, 150 μL/s) and taking a CCD image for 60 s.

### 2.7. Data Analysis

Images of CL microarrays were evaluated with the software MCRImageAnalyzer (GWK GmbH, Munich, Germany) as previously described [31]. The CL signal (CLS) was calculated by taking the ten brightest pixels of each of the five spots in a row. Significant outliers of CLS (deviation from the mean > 20%) were not included for the calculation. The efficiency of desorption (DE) was calculated with the ratio of CLS after injection of sera (CLS_S_) and CLS after denaturation of proteins (CLS_D_):

(1) $$DE=\frac{CLS_{S}}{CLS_{D}}$$

An efficient denaturation should result in values that are much higher than 1. The efficiency of regeneration (RE) was calculated using the following equation:

(2) $$RE=\frac{CLS_{S,n}-CLS_{D,n-1}}{CLS_{S,0}-CLS_{D,0}}\times 100\%$$

where *n* is the value of repeated regeneration per rAg microarray chip and number 0 means the first CLS values before regeneration.

## 3. Results and Discussion

The flow-based CL microarray immunoassay (CL-MIA) for detection of antibodies in sera of slaughtered pigs is based on an indirect antibody capturing method. A microarray of immobilized rAgs ORF2C-gt1, ORF2C-gt3, and YopD was used on the MCR3 that was able to detect antibodies against the zoonotic pathogens HEV and *Yersinia* spp. in swine sera.

Since there were no standards of swine antibodies available, two sera of slaughtered pigs were applied in this study phase. Each specimen tested positive for HEV using the *recom*Line HEV IgG/IgM kit (Mikrogen, Neuried, Germany), adapted for swine sera [24], and for *Yersinia* spp. using the pigtype Yersinia Ab kit (Qiagen Leipzig, Leipzig, Germany). Samples were collected at Bavarian slaughterhouses and processed at the Chair of Food Safety, Veterinary Faculty and LMU Munich. For rAg microarray experiments, sera were diluted 1:100 in PBST, filled into a 1-mL plastic syringe and placed in the injection unit of the MCR3. The CL-MIA was processed automatically with help of the fluidic system that is equipped with syringe pumps and valves. After 7 min, the captured antibodies on the immunochip were detected by HRP-conjugated polyclonal anti-swine antibodies and a following CL reaction. The localized CL reaction at each spot was recorded with a CCD camera. CLS_S_ of antibody capturing assays correlates directly with the concentration of antibodies in sera.

**Figure 3.**
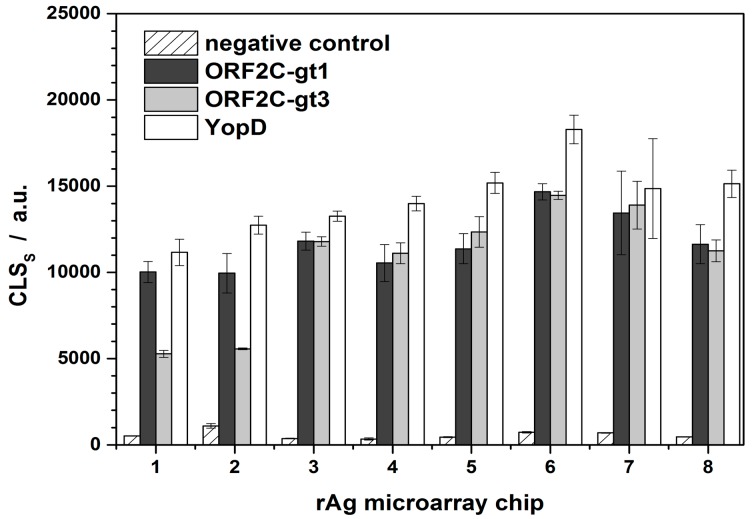
Results of HEV and Yersinia positive swine sera on 8 rAg microarray chips containing negative control and rAgs ORF2C-gt1, ORF2C-gt3, and YopD. Error bars indicate the standard deviation of five spots.

CLS_S,0_ were determined for each immunochip. A new rAg microarray chip was used for each series of regeneration experiments (Figure 3). For microarray chips 1–3 and 4–8, two different swine sera were used that were positive for ORF2C-gt1, ORF2C-gt3, and YopD with similar CL intensities, respectively. rAg microarrays 1 and 2 were prepared in one load and the others in a separate batch. This explains the significant difference of CLS_S,0_ regarding the rAg ORF2C-gt3 of microarray chips 1 and 2. The variances for the microarray chips were 13%, 30%, and 14% for ORF2C-gt1, ORF2C-gt3, and YopD, respectively. Low variances indicated that apart from the two first measurements of spotted rAg ORF2C-gt3, the preparation of rAg microarray chips was reproducible.

**Figure 4.**
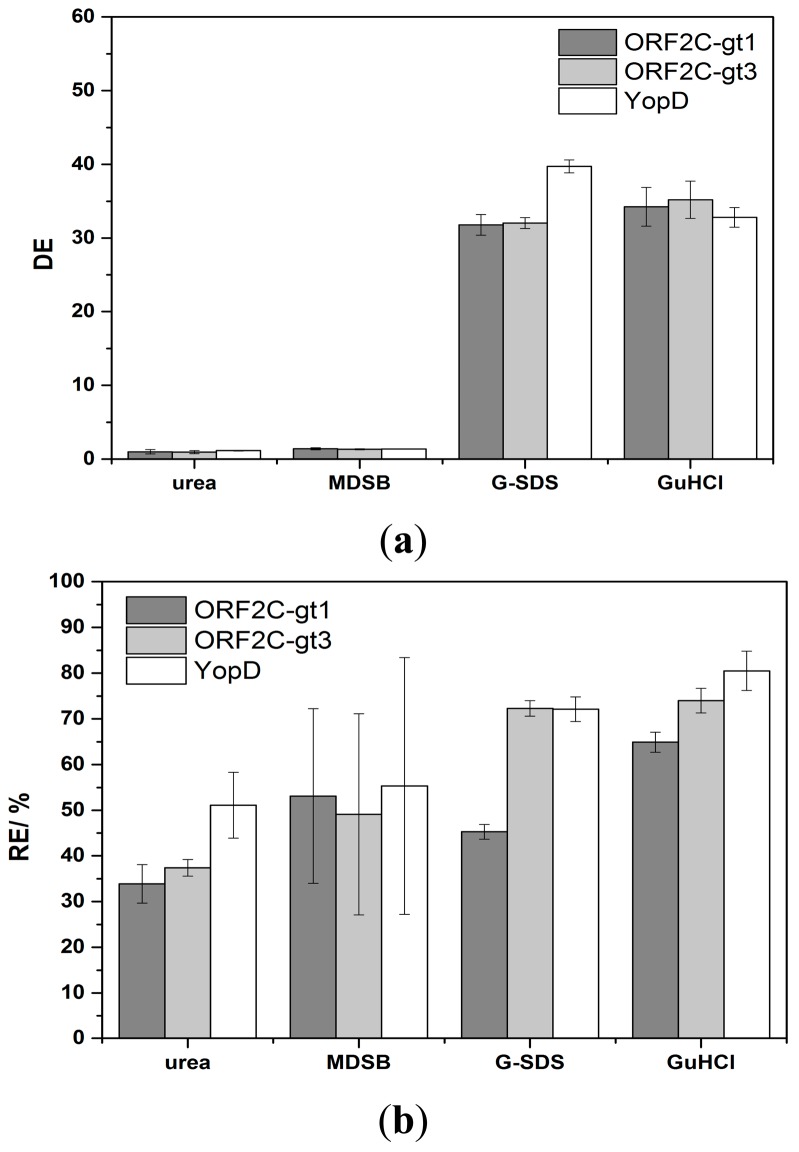
Results for denaturation and subsequent reactivation with RB1 of rAg microarrays. According to Equations (1) and (2), Section 2.7., (**a**) shows how effective the antibody complexes are desorbed by the denaturation buffer and (**b**) illustrates the effectivity of the whole regeneration process. Error bars indicate the standard deviation of five spots.

The regeneration of immobilized proteins was achieved by subsequent flow-based incubation with denaturation and reactivation buffers. The denaturation step should efficiently desorb sera antibodies and HRP-labeled detection antibodies. Concurrently, immobilized rAgs were denatured. A reactivation of immobilized rAgs was necessary because the native protein structure was important for the next binding step of antibodies in sera. High CLS_D_ shows that the antibody complexes were not efficiently desorbed and the enzyme activity of HRP was not significantly reduced. DE as a ratio of CLS_S_ and CLS_D_ was in this case near to 1. The interpretation of low CLS_D_ was more complex. Either HRP was inactive but the antibody complex was not desorbed or the complete antigen-antibody complex was desorbed. Non-desorbed antibody complexes would reduce CLS_S_ of the next measurement cycle. However, lower CLS_s_ would also occur if the reactivation process of rAgs was not sufficient or if the immobilized rAgs were desorbed. RE values were calculated as ratio of CLS_s_ between two measurement cycles and CLS_D,0_ and CLS_D,n-1_ is subtracted, respectively. The efficiency of desorption and reactivation was studied with four denaturation buffers (6 M urea, MDSB, G-SDS and 8 M GuHCl) and two reactivation buffers. 6 M urea is a standard denaturation buffer of proteins [32]. MDSB is used because the provided rAgs for immobilization on the microarray chip were stored denatured in this buffer. G-SDS is an acidic low salt buffer that contains SDS for denaturation of proteins and is often used for regeneration of flow-based immunoassays [26]. 8 M GuHCl is a chaotropic denaturation agent and is described in many protein preparation applications [33]. In a first study, rAgs were denatured with one of the four denaturation buffers, respectively, and reconstituted with RB1. Desorption of antibody complexes was not sufficient using 6 M urea or MDSB as denaturation buffer. However, an efficient desorption took place with G-SDS and 8 M GuHCl (Figure 4a).

Using urea, DE was 1.0, 1.0, and 1.2 for ORF2C-gt1, ORF2C-gt3, and YopD after the first measurement cycle, respectively. No increase of DE was achieved by repeating the regeneration process 10 times (data not shown). This result indicates that no desorption took place and the enzyme activity of HRP remained constant. For MDBS, the DE values were 1.4, 1.3, and 1.4 for ORF2C-gt1, ORF2C-gt3, and YopD after the first measurement cycle, respectively. DE was only slightly greater than 1 for desorption of antibody complexes with MDBS and no increase was determined by repeating 9 times the regeneration experiments (data not shown). For G-SDS, the DE values were 31.8, 32.0, and 39.7 for ORF2C-gt1, ORF2C-gt3, and YopD after the first measurement cycle, respectively. GuHCl shows with values of 34.3, 35.2, and 32.8 for ORF2C-gt1, ORF2C-gt3, and YopD similar desorption efficiencies.

Considering RE values, 30%–50% of the CL signal remained for 6 M urea and MDSB (Figure 4b). RE was lower than 6% after nine measurement cycles for both denaturation buffers and all rAGs (data not shown). This fact indicates that desorption was not efficient and the binding sites on rAGs for antibody capturing were stepwise reduced. 6 M urea and MDBS were not sufficient for regeneration of rAg microarrays. Higher RE values were received with G-SDS and GuHCl. RE was 45.3%, 72.3%, and 72.1% for ORF2C-gt1, ORF2C-gt3, and YopD for G-SDS, respectively. Slightly higher results were obtained with GuHCl. RE was 64.9%, 74.0%, and 80.5% for ORF2C-gt1, ORF2C-gt3, and YopD, respectively.

**Figure 5.**
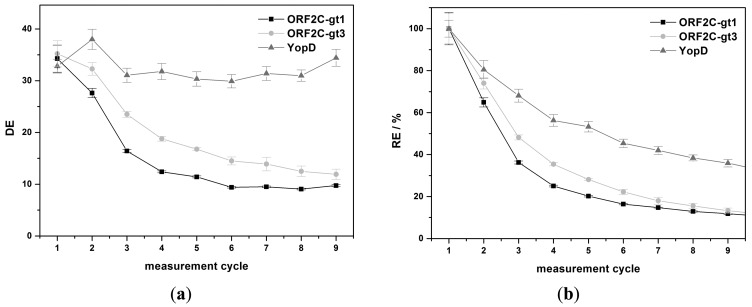
Results for denaturation with 8 M GuHCl and subsequent reactivation with RB1 of rAg microarrays. According to Equations (1) and (2), Section 2.7, (**a**) shows how effective the antibody complexes are desorbed by the denaturation buffer and (**b**) illustrates the effectivity of the whole regeneration process. Error bars indicate the standard deviation of 5 spots.

**Figure 6.**
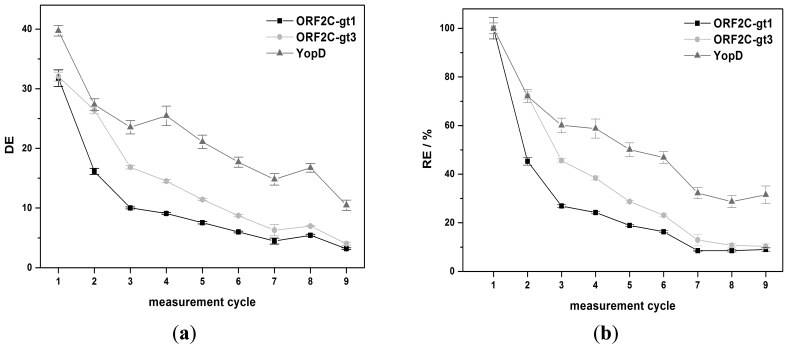
Results for denaturation with G-SDS and subsequent reactivation with RB1 of rAg microarrays. According to Equations (1) and (2), Section 2.7., (**a**) shows how effective the antibody complexes are desorbed by the denaturation buffer and (**b**) illustrates the effectivity of the whole regeneration process. Error bars indicate the standard deviation of five spots.

The regeneration behavior was studied over nine measurement cycles for 8 M GuHCl and G-SDS. 8 M GuHCl shows nearly a constant DE value (32.3% ± 2.4%) for YopD (see Figure 5a). An exponential decrease of DE was observed for ORF2C-gt1 and ORF2C-gt3. DE was reduced by 71.6% and 66.2% after nine measurements for ORF2C-gt1 and ORF2C-gt3, respectively. The RE values were reduced to 10.6% and 11.5% for ORF2C-gt1 and ORF2C-gt3, respectively. However, RE of YopD was as well reduced to 35.9% after nine measurements (see Figure 5b). This result suggests that immobilized rAgs were partly desorbed and YopD was not reactivated completely after the subsequent treatment with GuHCl and RB1.G-SDS shows an exponential decrease of DE for all three rAgs (see Figure 6a). DE was reduced by 90.0%, 87.5%, and 73.7% after nine measurements for ORF2C-gt1, ORF2C-gt3, and YopD, respectively. As shown in Figure 6b, the RE values were reduced to 8.5%, 10.7%, and 28.7% for ORF2C-gt1, ORF2C-gt3, and YopD, respectively. The regeneration of rAgs was therefore similar for GuHCl and G-SDS. Taking into account that GuHCl is very expensive and high salt concentrations destroy the flow system for routine usage, G-SDS was the most suitable denaturation buffer for regeneration of rAgs in this study. The reactivation can be slightly improved by using RB2 (0.1% (w/v) BSA) instead of RB1 (0.05% (v/v) Tween^®^-20). Determined RE values after nine measurements with RB2 were 15.8%, 16.0%, and 44.2% for ORF2C-gt1, ORF2C-gt3, and YopD, respectively.

**Figure 7.**
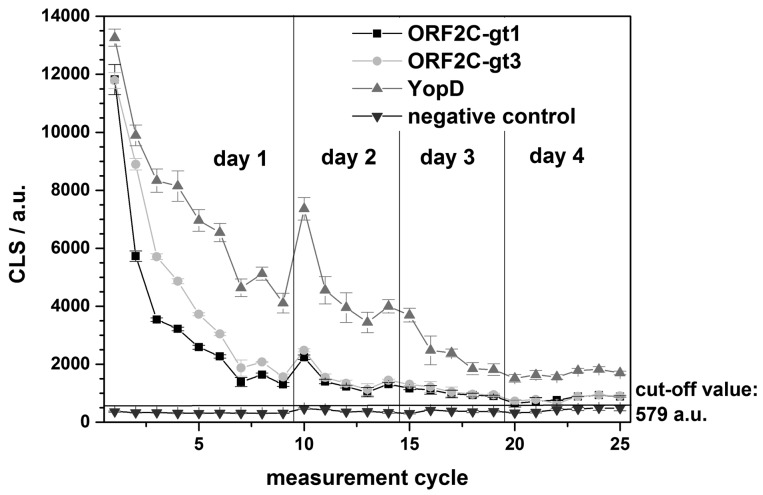
Results for regeneration experiments over 4 days on one rAg microarray chip with G-SDS as denaturation buffer and RB1. Error bars indicate the standard deviation of five spots.

The regeneration of one rAg microarray chip was repeated over 4 days for a total of 25 measurements. A single sample reactive for antibodies to both HEV and *Yersinia* spp. was tested using G-SDS and RB1. An internal negative control was used to determine the chip cut-off value (mean of the negative control + 10 times the standard deviation). In this case, the cut-off value for the chip was: 373 a.u. (mean of the negative control) + 10 × 21 a.u. (standard deviation) = 383 a.u. Using this cut-off, the reactive sample remained antibody-positive for the three rAgs on the microarray for all 25 measurements over the 4-day testing period (Figure 7). Overnight storage at 4 °C between testing days appeared to have no effect on chip performance.

## 4. Conclusions

A method for studying the regeneration of rAg microarrays was successfully established for the flow-based MCR3 CL microarray analysis platform. The CLS values measured after the complete regeneration process could be misinterpreted because non-desorbed active HRP conjugates could also result in high CLS values. G-SDS was found as the best denaturation buffer based on the remaining CLS_s_ and the low CLS_D_. G-SDS is efficient and economical. GuHCl also shows a substantial denaturation effect but the high salt concentration of the buffer could damage the flow system, and the costs for this chemical product are high. Reconstitution of immobilized rAgs was possible using carbonate buffer containing 10 mM DTT and either 0.01% (v/v) Tween^®^-20 (RB1) or 0.1% BSA (RB2).

CLS values gradually decreased because some of the immobilized rAgs were desorbed from the microarray surface. Although rAgs are primarily immobilized by covalent bonds between primary amino groups and the epoxy-activated chip surface, over-saturation might occur due to a high concentration of rAgs in the spotting solutions. Thus, further experiments will be required to optimize these concentrations.

However, significant positive results for antibodies against HEV and *Yersinia* spp. were obtained with the regenerated immunochips over 4 days (*n* = 25). A rapid and fully automated analysis of antibodies to multiple infectious agents in sera of slaughtered pigs may be possible in the future. These results are important for the continued development of microarray-based immunoassays to monitor zoonotic pathogens in the meat processing industry.

## Abbreviations

BSA: Bovine serum albumin
CL: Chemiluminescence
CL-MIA: Chemiluminescence microarray immunoassay
CLS: Chemiluminescence signal
DAPEG: Poly(ethylene glycol) diamine
DE: Efficiency of desorption (calculated according to Equation (1), Section 2.7.)
diepoxy-PEG: Poly(ethylene glycol) diglycidyl ether
DTE: Dithioerythritol
DTT: Dithiothreitol
GOPTS: 3-Glycidyloxypropyl trimethoxysilane
GuHCl: 8 M guanidinium hydrochloride
G-SDS: Buffer containing 100 mM glycine, 100 mM NaCl, and 1% SDS (pH 2.5)
HEV: Hepatitis E virus
HRP: Horseradish peroxidase
IgG: Immunoglobulin G
MCR3: Munich chip reader 3rd generation
MDSB: Buffer containing 20 mM MOPS, 10 mM DTT, and 0.1% SDS
MOPS: 3-(*N*-morpholino)propanesulfonic acid
MTP: Microtiter plate
ORF2C-gt1/gt3: Recombinant open reading frame 2C antigen of HEV genotype 1 and 3
PBST: Phosphate buffered saline containing 0.05% Tween^®^-20
rAg: Recombinant antigen
RB: Reactivation buffer
RE: Efficiency of regeneration (calculated according to Equation (2), Section 2.7.)
SDS: Sodium dodecyl sulfate
TRIS: Tris(hydroxymethyl)aminomethane
YopD: Recombinant Yersinia outer protein D antigen of *Yersinia* spp
